# Acupuncture and Neural Mechanism in the Management of Low Back Pain—An Update

**DOI:** 10.3390/medicines5030063

**Published:** 2018-06-25

**Authors:** Tiaw-Kee Lim, Yan Ma, Frederic Berger, Gerhard Litscher

**Affiliations:** 1 University Postgraduate Education of Principles and Practice of Traditional Chinese Medicine, Medical University of Vienna, 1090 Vienna, Austria; jalimee@gmail.com (T.-K.L.); yan.ma@meduniwien.ac.at (Y.M.); 2 Institute of Pathophysiology and Allergy Research, Center for Pathophysiology, Infectiology and Immunology, Medical University of Vienna, 1090 Vienna, Austria; 3 Gregor Mendel Institute of Molecular Plant Biology GmbH, Austrian Academy of Sciences, 1030 Vienna, Austria; frederic.berger@gmi.oeaw.ac.at; 4 Research Unit of Biomedical Engineering in Anesthesia and Intensive Care Medicine, Research Unit for Complementary and Integrative Laser Medicine, and TCM Research Center Graz, Medical University of Graz, 8036 Graz, Austria

**Keywords:** low back pain (LBP), acupuncture, mechanism of acupuncture, anti-nociceptive, purinergic receptors, adenosine triphosphate (ATP), adenosine

## Abstract

Within the last 10 years, the percentage of low back pain (LBP) prevalence increased by 18%. The management and high cost of LBP put a tremendous burden on the healthcare system. Many risk factors have been identified, such as lifestyle, trauma, degeneration, postural impairment, and occupational related factors; however, as high as 95% of the cases of LBP are non-specific. Currently, LBP is treated pharmacologically. Approximately 25 to 30% of the patients develop serious side effects, such as drowsiness and drug addiction. Spinal surgery often does not result in a massive improvement of pain relief. Therefore, complementary approaches are being integrated into the rehabilitation programs. These include chiropractic therapy, physiotherapy, massage, exercise, herbal medicine and acupuncture. Acupuncture for LBP is one of the most commonly used non-pharmacological pain-relieving techniques. This is due to its low adverse effects and cost-effectiveness. Currently, many randomized controlled trials and clinical research studies have produced promising results. In this article, the causes and incidence of LBP on global health care are reviewed. The importance of treatment by acupuncture is considered. The efforts to reveal the link between acupuncture points and anatomical features and the neurological mechanisms that lead to acupuncture-induced analgesic effect are reviewed.

## 1. Introduction

Low back pain (LBP) is one of the most frequently encountered musculoskeletal disorders in today’s society. LBP is defined as pain and discomfort, located in between the costal margin and the inferior gluteal folds, with or without referred leg pain [1,2,3,4]. LBP is categorised according to duration as acute (less than 6 weeks), sub-acute (between 6 and 12 weeks), or chronic (more than 12 weeks) [4,5,6].

LBP interferes with activities of daily living [7], work performance [8,9], and is a major reason for people to seek medical consultation [10,11]. This disorder contributes to a substantial burden on individuals, employers, the healthcare system and society in general. According to a report published by the World Health Organization (WHO) in 2013, back pain, together with neck pain, was the second highest cause amongst the 20 leading non-fatal health outcomes from the year 2000 to 2011 [12].

The survey, The Global Burden of Disease Study 2016 (GBD 2016), published in the Lancet in 2017 [13,14], highlights the extent of health loss due to diseases, injuries, risk factors, prevalence and mortality rate by age, sex, and geography at specific points in time. In the GBD 2016, LBP was the number one cause for the most years lived with disability (YLDs) in the world [13].

The disability-adjusted life years (DALYs) are calculated as the combination of years of life lost (YLLs) due to premature mortality and years lived with disability (YLDs) [14,15]. Because LBP does not cause mortality, therefore YLDs are the same as disability-adjusted life years (DALYs). DALYs for LBP, together with neck pain, was on the 4th position out of the 30 leading global DALYs in the GBD 2016 [14]. This was much higher than the average DALYs related to road injuries, HIV/AIDS, diabetes, chronic obstructive pulmonary disease (COPD) and lung cancer [14]. The overall estimation of DALYs for LBP in 2016 was 57.6 million, which represents more than 40% of the total number of 140 million DALYs of all the musculoskeletal disorders combined. In Austria, the GBD 2015 for YLDs and DALYs due to LBP and neck pain are ranked at No. 1 and No. 2, respectively [16].

The estimated global prevalence of the population affected by LBP was 9.2% according to WHO in 2010 [12]. Hoy et al. estimated that the ranges of prevalence of LBP at a point, 1-month, 1-year and over a lifetime were 18.3%, 30.8%, 38.0% and 38.9%, respectively [17]. In the GBD 2016, it was estimated that the prevalence of LBP for the year 2016 was more than 511 million of the world’s population, an increase of 18.0%, as compared to 2006 [7]. LBP results in high costs to society due to increased demands on the healthcare system and work absence. In the USA alone, it has been estimated that LBP costed between US $100–200 billion yearly [1]. This amount has more than doubled from 1991 to 2016 [18].

In this scientific article, causes of LBP are surveyed and the links between anatomical features and acupuncture points are outlined. This is followed by a review of randomized controlled trials (RCTs) and of mechanisms potentially targeted by acupuncture treatments.

## 2. Causes and Risk Factors of Lower Back Pain

LBP is very common, and yet is a very complex multifactorial disorder with much possible etiology. These originate from injuries, trauma or fractures to the anatomical structure [19,20,21]; lumbar spine degeneration [22,23]; and disc herniation or nerve entrapment [24,25,26]. Other causes associated with an increased risk of LBP, include infections, autoimmune diseases, orthopedic diseases or tumours [27,28,29,30,31,32,33]. LBP results also from occupational ergonomic factors related to heavy physical work, repetitive actions due to occupational requirements [1,2,34,35,36,37,38,39]; as well as sports activities or sports-related injuries [40,41]; sedentary lifestyle, prolonged sitting or inactivity and lack of exercises [42,43,44]; post operation or surgery-induced [45,46]; secondary from other medical conditions [47,48,49]; lifestyle factors [50]; poor trunk control and postural impairment [51]; psychosocial and behavior-related factors from smoking, alcohol abuse, obesity, depression and stress [1,6,14,52,53,54,55,56,57]; socio-economic factors [58]; and ageing [59,60]. However, it is often difficult to identify the origin of LBP [1,5,6,61,62,63,64,65] and 85–95% of the total cases of back problems [1,63,64,65] are not associated with a specific patho-anatomical origin, or attributed to any recognizable pathology patterns. 

### 2.1. Age and Gender

The age group 40–69 is affected with the highest incidence of LBP [17], and women have higher LPB prevalence than men [17,50,57,66,67,68,69].

Großschädl et al. studied the LBP prevalence of the Austrian population in 1983, 1991, 1999, and 2006/7. During this period, they found a marked increase in the rate of LBP for both sexes, especially more so in women over recent years [69]. This increasing trend of the rate of LBP is recorded worldwide and might be related to women’s involvement in the workforce, while their house chores do not diminish [69,70]. LBP’s risk factors specific to women comprise exposure to musculoskeletal loads due to pregnancy [71,72,73]; menstruation [74,75]; menopausal [66,76,77]; hormone [66,78]; osteoporosis [68,79]; low bone mineral density [80,81]; and conditions associated with ageing [59,82]. In contrast, certain studies show that men have a higher rate of LBP [1,2], especially those involved in heavy physical work and repetitive movements [1,2,6,38,39].

### 2.2. Obesity and Smoking

Schneider et al. reported that the prevalence of LBP is significantly higher for women affected by overweight, low level of social support, a sedentary lifestyle, smoking and lower income groups [67]. The association between obesity and LBP remains controversial. Few studies on the relation between obesity and LBP have been published so far. Obesity is considered as one of the elements that contributes to the increasing rate of LBP [56,57,67,68,69,83,84]. Heuch et al. estimated that the rate of LBP increases in parallel with the rate of body mass index (BMI) [83]. As stated by a guideline by WHO, an adult is viewed as overweight when the BMI is greater or equal to 25, and one is considered obese when the BMI has reached 30 or more [85]. In addition, increased BMI also affects the incidence of neck pain and arthritis [86]. A group of studies led to speculation of connections between smoking and obesity that may result in LBP [67,87]. Yet, such a conclusion was challenged by another study, which did not find such link between LBP, smoking and obesity [88].

## 3. Theories of Pain

For many centuries, people have been seeking the origin of pain and ways to alleviate it. Despite a marked progress in our understanding of pain, many aspects of the mechanisms involved remain unclear. Pain is a very subjective issue, as it is interpreted differently by each patient. Some people have a very high pain threshold, which allows them to tolerate pain extremely well. For others, even a slight trigger of stimulation might cause them to suffer in agony. Because of this huge difference in perception of pain magnitude, it is difficult to understand the degree of suffering by a patient. The visual analogue scale (VAS), which was created in 1921 by Hayes and Patterson [89], is a simple tool to judge a patient’s perspective of pain, to translate a subjective level of pain into an objective measurement. 

Pain is classified as a nociceptive pain (caused by excitation of nociceptors by external stimuli), inflammatory pain (intervened by inflammatory mediators released by an inflamed organ), or neuropathic pain (induced by lesions of the central or peripheral nervous system) [90]. According to Merskey and Bogduk, “pain is an unpleasant sensory and emotional experience associated with actual or potential tissue damage or described in terms of such damage” [91].

The discovery of nociceptor by Charles Sherrington in 1906, has changed forever the way we consider the concept of the central nervous system (CNS) [92]. This gave rise to many modern studies about pain management, such as nerve blocking and analgesic effect by acupuncture. The Gate Control Theory of Pain, which was proposed in 1965 by Melzack and Wall, suggested that the brain has a “gate” mechanism either blocking or allowing pain messages to reach the brain [93]. Although their theory was later proven to be flawed, it did provide a useful overall concept of how pain is experienced and many researchers engaged in this field of study. One of them resulted in the invention of transcutaneous electric nerve stimulation (TENS) for the management of LBP [94].

In 1973, Pert and Synder discovered the opiate receptor, the cellular binding site for endorphins in the brain [95]. Two years later, a group of British scientists led by Hughes and Kosterlitz, found the breakthrough in the field of molecular receptor studies, the discovery of enkephalin [96]. This was followed by studies that led to our current knowledge of endorphins, the body’s very own natural mechanism of painkiller [97,98,99,100].

To be able to ease the pain experienced by patients, first we need to understand the root cause of pain and the mechanism that triggered the transmission of pain. Having a better understanding of the nervous system’s own mechanism of analgesia, such as that triggered by the stimulation of needles by acupuncture, pain management of LBP by acupuncture is no longer a placebo or a contextual effect, but rather, an evidence-based scientific proof of an ancient practice. These topics are addressed in more detail below.

## 4. Pain Mechanism

The mechanism of pain is a very complex process, due to the involvement of multiple layers of the neural circuit, from the stimulation of the receptors to the chemical reaction in the CNS. The perception of pain is called nociception, and it is initiated by nociceptors present at free nerve endings [101]. The nociceptors, part of the architecture of neural circuits, are spread all over the body, from the superficial layers of skin to the deeper tissues and internal organs.

Tissue injury by noxious stimuli is detected by the nociceptors. This information is transmitted as electrical nerve impulses, or action potentials, via the nerve fibres, called the primary afferent neurons to the dorsal root ganglia (DRG). DRG are linked to dorsal root in the spinal cord through the dorsal root. The electrical signal synapses with the second afferent neuron at the dorsal horn of the grey matter. From here, the signal crosses over to the opposite side of the spinal cord, and connects to the ventral white matter and further links to the spinothalamic tract. The signal is now traveling upward from the spinal cord to the thalamus in the brain where pain is generated. In the thalamus, a third synapse occurs and the nerve impulse is transmitted via the thalamocortical tract to the cerebral cortex. This process tells us the exact location of the pain. Nociceptors enable humans to recognise pain stimuli and therefore to respond accordingly, such as pulling away our hand from a fire or sharp object [102,103,104,105,106,107,108,109].

### 4.1. Nerve Fibres

There are two types of nerve fibres, afferent and efferent. Afferent means ascending, and qualifies sensory fibres sending signals to the brain. While efferent signifies descending, and defines motor fibres, which relay messages away from the CNS. The afferent nerve fibres convey signals concerning potential damage or injury from outside and inside the body. 

The afferent nerve fibres in the human’s body are further divided into four major types of primary sensory neurons, which can be differentiated morphologically and functionally into A-alpha (Aα), A-beta (Aβ), A-delta (Aδ) and C fibre [110]. Aα fibres represent motor fibres connected to voluntary muscles and include certain sensory fibres that transmit position sensation from skeletal muscles. Aβ fibres carry non-noxious stimuli and convey the sensations of touch, vibration and pressure from the skin [111]. There are only two types of nerve fibres involved in the transmission of painful impulses. The Aδ fibre is myelinated, enables fast transmission of impulses and produces sharp and well localised pain. The second type, the unmyelinated C fibre, is slow in transmission and produces dull or burning pain, the exact location of which is diffused and poorly localised [105,106,107,109]. Kagitani et al. demonstrated that acupuncture stimulation enables production of various autonomic functions on both Aδ and C fibres [110]. While Zhao suggested that stimulation by manual acupuncture (MA) and electroacupuncture (EA) activates Aδ and C fibres to produce an analgesic effect [112].

### 4.2. Inflammatory Soup

Tissue damage causes release of a variety of chemical substances into the extracellular space around the receptor terminals. These chemical substances comprise bradykinin, histamine, serotonin, prostaglandins, nerve growth factor (NGF), substance P, adenosine triphosphate (ATP), calcitonin gene-related peptide (CGRP), protons (H⁺) and other purines and indolamines. Altogether, they form the “inflammatory soup” and are able to interact and activate the nociceptive fibres that cause localised pain and inflammation [102,103,104,105,106,107,108,109].

## 5. Treatment of LBP by Acupuncture

The goal of LBP treatment is to control or reduce pain, to improve structure impairment of the spine and to return to the normal life activities as soon as possible. Most current international guidelines recommend pharmacological management for pain relief of LBP, including paracetamol, non-steroidal anti-inflammatory drugs (NSAIDs), muscle relaxant, opioid analgesics, epidural steroid, anticonvulsants, antidepressants, and corticosteroids, among others [1,5,61,65,113,114,115,116]. However, most of these pharmacological treatments produce limited pain relief and are accompanied by serious side effects, such as drowsiness, dizziness, addiction, allergic responses, reversible reduction of liver function, and negative impacts on gastrointestinal functions [1,5,61,113,114,116,117]. At least one of these side effects is experienced in approximately 25 to 30% of patients treated with opioids [118]. The major problem with this approach is that pain may be temporarily relieved but the source of LBP is not identified and alternative treatments of LBP are required. These include multidisciplinary rehabilitations based on physiotherapy, spinal manipulation, exercise therapy, massage therapy, cognitive-behaviour therapy, yoga, tai-chi, and acupuncture [5,61,65,113,115]. Here, the focus is on a critical assessment of the benefits of acupuncture in LBP treatment.

### 5.1. Acupuncture

Acupuncture is part of the healing system of Traditional Chinese Medicine (TCM). It consists of insertion of thin needles into the muscle, on specific acupuncture points placed along meridians to treat a variety of conditions. Apart from the traditional manual acupuncture (MA), there are other methods to stimulate acupuncture points for therapeutic purposes, such as electroacupuncture (EA), acupressure, laser acupuncture and moxibustion.

The practice of acupuncture as pain relief is widely used in many of the countries throughout the world [119]. Despite a lack of well-designed clinical studies supporting its efficacy, and skeptical opinions from many, it is nevertheless well accepted by many patients globally. Acupuncture is legally recognised by many countries in Asia, Australia, America, Canada, and some parts of Europe and Latin America [120].

World Health Organization (WHO) estimated that one-third of the world’s population has no regular access to modern medicines, especially in many parts of Africa, Asia and Latin America. Fortunately, complementary and alternative medicine (CAM) such as herbal and traditional medicine and acupuncture are available and accessible [120].

In the USA alone, the number of acupuncture users increased by 50% between 2002 and 2012 [121]. As of December 2010, there were approximately 305,000 registered Complementary & Alternative Medicine (CAM) providers across Europe, including 96,380 acupuncturists. Eighty-thousand were medical and 16,380 were non-medical practitioners [122]. In Austria, there were 3531 doctors qualified with the diploma of acupuncture from the Austrian Medical Association (Diplom für Akupunktur der Österreichischen Ärztekammer) in 2011 [123]. According to the Law of Health Services Austria (Bundesgesetz über die Gesundheit Österreich), acupuncture is a scientifically recognized treatment that can be provided in hospitals [124]. In contrast with increasing interest and practice of acupuncture, there is a persistent lack of infrastructure and funding dedicated to research and teaching in this field [125].

### 5.2. Brief History of Acupuncture

The root of Traditional Chinese Medicine (TCM) can be traced back over 3000 years ago in China. While acupuncture is the best known modality of TCM in the West, TCM has a long history of great use of herbal remedies. Apart from acupuncture and herbal medicine, TCM also comprises of Qigong, Taichi and Tuina. The first written medical text on acupuncture was mentioned in the Huang Di Nei Jing (The Yellow Emperor’s Internal Classic). It comprised of two volumes: The first one is Su Wen (Basic Questions), which covers the diagnostic methods and theoretical foundation of TCM [126]. The second part is called Ling Shu (The Spiritual Pivot) and explains acupuncture therapy such as the description of meridians, functions of the acupuncture points, needling techniques, types of Qi and the location of 160 points [127]. Two other classic texts which mention acupuncture in depth are Nan Jing (The Classic of Difficult Issues) [128] and Zhen Jiu Jia Yi Jing (The Systematic Classic of Acupuncture and Moxibustion). The latter was written by Huangfu Mi, who added another 189 acupuncture points to reach a total of 349 [129]. The precise history of these four books is unclear as they were edited, annotated and reinterpreted several times. Another obstacle of learning and understanding the teaching of TCM is the difficulty in translating these ancient Chinese texts [130]. 

Unlike western medicine, which has a clear distinct path that can be traced back to Hippocrates [131], there is undocumented evidence to when and where the origin of acupuncture came from due to the loss of many of the valuable ancient texts. The archaeological discovery of “bian shi” (a kind of flattened and sharpened stone) from the era of the New Stone Age in China (8000–2000 BC), was believed to represent the ancient acupuncture needle for treating illnesses by pricking certain parts of the body. During the Warring States (475–221 BC), metal needles were developed to substitute the stone needles [132]. 

In the 6th century, acupuncture together with the teaching of TCM spread to Korea and Japan. Later in the 10th century, acupuncture arrived in Vietnam through the commercial routes. The practice of acupuncture was brought back to France by the Jesuit missionaries in the 16th century [133]. The very first acupuncture description that appeared in the West was written by Wilhelm Ten Rhyne in 1683, a physician employed by the Dutch East India Company, who was based in Japan [134].

In the USA, acupuncture has gained sudden attention and popularity thanks to the New York Times writer James Reston’s article in 1971, entitled “Now, About My Operation in Peking” [135]. He described his first-hand experience of acupuncture with an emergency appendectomy and post-operative care while on his assignment to China prior to President Nixon’s visit. This has prompted many doctors visiting China to observe and study the effectiveness and benefits of acupuncture, with a specific interest in its use for surgical analgesia [136,137,138,139,140].

### 5.3. Theory of Traditional Chinese Medicine

Traditional Chinese Medicine (TCM), one of the most important components of Chinese culture, has a long-established history and consists of a wealth of experience and a profound source of knowledge. It has made a great contribution to the overall welfare of its people. At present, TCM is also becoming well known globally, playing a unique role in the development of public healthcare [141].

In contrast to the anatomical and scientific perspective, the theories of the mechanism of action in TCM on LBP differ significantly from those of modern pharmacology. TCM states that pain is caused by internal disharmony between Yin and Yang, imbalance between Qi and Blood, and disparity within the Five Elements [142]. Qi, the essence of life, is believed to circulate within special conduits in the body termed meridians and collaterals that connect all parts of the body, including connecting the organ systems with each other and their related sense organs. When there is insufficiency of Qi, or when the meridians are blocked, this causes an imbalance between Yin and Yang, perturbs the meridians, hinders the smooth flowing of Qi and results in stagnation of Qi and blood, leading to pain and illness [143,144,145,146].

## 6. Acupuncture Principles

Acupuncture is based on the theory of channels, simply referred to as meridians and collaterals, which are the branches of the meridians. There are 14 main meridians, and each consists of an internal pathway that runs inside the body and links to an external pathway where acupuncture points are located. Qi flows through these pathways of meridians interconnecting with each other, and is linked to specific internal organs, which are also called Zangfu organs [146]. Stimulation on the acupuncture points by acupuncture needle enables unblocking of the meridians, hence promoting a smooth flow of Qi [147].

### 6.1. The Anatomical Structure of Acupuncture Points and Meridians

Detailed lists and definition of acupuncture points are provided in several textbooks [148,149,150,151]. Deadman et al., in their book, A Manual of Acupuncture [151], mentioned that “centuries of observation of the existence of tender spots on the body during the course of disease, and the alleviation of symptoms when they were stimulated by massage or heat, led to the gradual discovery of the acupuncture points”. This sheds a bit of light on the origin of acupuncture. The meridians and the acupuncture points can be compared with airline travel routes: They exist on a map but cannot be “seen”.

Many studies have been carried out to obtain concrete evidence supporting the existence of acupuncture points and the Qi flowing meridians. Back in 1963, the North Korean scientist, Kim Bong Han, discovered the Kyungrak System (it means acupuncture meridians and collaterals in Korean), better known as the primo vascular system (PVS). PVS is a threadlike structure defining a circulatory system entirely different from the vascular, nervous, and lymphatic systems. Kim established that the PVS represents anatomical structures linked to acupuncture points and meridians. He proposed that the PVS has the ability of hematopoietic functions as well as to regenerate injured tissues and heal wounds [152]. Many scientists tried to reproduce his result but failed. That was because Kim did not mention what kind of staining blue dye was used in his studies [153]. In 2002, Shin et al. confirmed the existence of PVS [154].

Stefanov et al. proposed that the PVS is the communication system between living organisms and the environment. They believed that this system is involved in channeling the flow of energy and information relayed by biophotons (electromagnetic waves of light) and is closely related to DNA [155]. Independent experiments involved injection of radioactive tracers at acupuncture points to attempt to map out and visualize the routes of meridians [156,157,158]. An alternative study by Langevin and Yandow led to the hypothesis that networks of interstitial connective tissue constitute the link between acupuncture points and meridians tissue [159]. Ultrasound imaging reveals a visible connective tissue forming an intramuscular cleavage plane at acupuncture points but not at control points [159]. Another anatomical distinctive feature of acupuncture points is high densities of nerve endings [160,161].

Other studies have explored the possibility of physiological differences between acupuncture points and surrounding tissues. Several reports conclude that the skin electrical impedance along meridians is lower than in their surroundings [162,163,164]. However, Stux and Pomeranz were quite skeptical about these kinds of electrical properties [165] and Kramer et al. concluded that skin electrical resistance at acupuncture points can either be lower or higher compared to the surrounding area [166]. In 2010, Litscher et al. developed a miniaturized 48-channel skin impedance measurement system for needle and laser acupuncture [164]. This system was further improved and a new multi-channel skin resistance measuring system, called GEDIS (Graz ElectroDermal Impedance measurement System) was used to differentiate the electrical skin resistance measurement between an acupuncture point and a placebo point. The system performs “electrodermal mapping”, and shows that the skin resistance at the acupuncture point has lower impedance values as compared to the non-acupuncture point [167]. 

Other studies suggested that there are more mast cells distributed at acupuncture points [168,169,170]. Marcelli, Peuker and Cummings, and Cheng, have made some studies of the morphological characteristics between the meridians and the anatomical structure [171,172,173,174]. Shaw and McLennan did an anatomical dissection study based on investigation of cadaveric specimens. They concluded that acupuncture points and meridians are purposefully named to reflect the observable physical form, e.g., heart meridian, lung meridian, etc. [175]. Anatomical dissections in ancient China are mentioned in chapter 12 of the Huang Di Nei Jing Ling Shu. Here, the imperial physician Qi Bo explains the fundamental structures of the human organism to the emperor Huang Di. The physician says: “After someone has died his body can be anatomically dissected and be examined (for medical investigation)” [127]. As another source of evidence to prove that acupuncture points are anatomically correct, the chapter 42 of Nanjing describes the length, diameter, weight and capacity of the internal organs [120]. In chapter 7 of Book 3 from Mi Huang-Fu’s Jia Yi Jing, the measurement of bones and intestines, and the volume of the stomach and intestines were clearly explained [129]. That is why the names for the internal organs used today are the same as the name of TCM organs defined more than 2000 years ago from Huang Di Nei Jing Ling Shu [127].

Acupuncture points are thus positioned at precise anatomical structures. For example, in TCM theory, the lung (LU) meridian is paired with the large intestine (LI) meridian [146]. LU 7 Lieque (Broken Sequence) is also the Luo-Connecting Point of the Lung meridian. From LU 7, the meridian branches out and links to the large intestine meridian, at LI 4 Hegu (Joining Valley), also known as Yuan-Source Point [151]. From an anatomical point of view, both points are located at the branch from the main cephalic vein, which is supplied by the radial nerve [176]. This evidence supports that acupuncture points and meridians correspond to anatomically defined positions in the human body. Pushing this idea further at the molecular level, Zhang et al. studied the connection between the heart and stomach meridians to cardiovascular diseases (CVDs) and gastrointestinal disorders (GIDs) and found that both CVDs and GIDs express sets of genes which are functionally related [177].

According to Thomas Myers, the author of Anatomy Trains, the whole body is a unique linkage of myofascial and locomotor anatomy in which the muscles are connected to the fascial net. The fascia, with an appearance akin to a spider’s web, is a continuous sheath of tissue, covering and interpenetrating every muscle, bone, nerve, artery, vein and lymph vessel, as well as, all internal organs, brain and spinal cord [178]. The fascial anatomy has a striking anatomical correlation to acupuncture points and meridians. Finando and Finando suggest that all fundamental characteristics of acupuncture treatment are consistent with stimulation of the fascia [179].

### 6.2. Acupuncture Points, Ashi Points, Myofascial Trigger Points (mTrPs) and Referral Pain

Interestingly, acupuncture points were rediscovered by researchers in western medicine. A study done by Kellgren in 1938 reported that intramuscular injection of sterile saline caused pain at locations away from the injection site [180]. Inspired by this study, Travell et al. realized that pressure applied to specific points (later defined as trigger points) relieved pain from shoulder and arm pain [181]. Later, Travell together with Simons, through their collaboration published their texts Myofascial Pain and Dysfunction, the Trigger Point Manual in 1983 [182,183]. Trigger points were described as locally tender points associated with focal areas of muscle shortening termed “taut bands”. When palpated, trigger points can feel like small nodules within the muscle and may refer pain distally [182,183]. Dorsher, did a study based on Travell and Simons’ texts, and found that 93.3% of the common myofascial trigger points (238 out of 255) correspond to the classical acupuncture points [184].

Translated into Chinese medicine terms, the above statement means that a trigger point is an area of Qi stagnation in the muscles. It also means that emotional stress may be the aetiological factor causing Qi stagnation in the muscles [144]. Ashi points, are very comparable to trigger points and were first mentioned in Bei Ji Qian Jin Yao Fang (Important Formulas Worth a Thousand Gold Pieces for Emergency) compiled by Sun Simiao in the Tang dynasty [185]. Chapter 13 of Huang Di Nei Jing Ling Shu, describes a treatment method, referring to Ashi points as “tenderness was taken as needling point” [127]. Ashi points are also used in other methods of treatment, such as Japanese shiatsu, Thai massage and Swedish deep tissue massage [186].

It is interesting to note that the route of referred pain from the heart, such as myocardial ischemia, is identical to the pathway of the heart meridian [187,188]. This kind of referred pain occurs due to the sharing of information between visceral and somatic afferent nerve fibres within overlapping spinal cord segments. The confusion about the origin of pain results from the density of sensory nerves higher in the skin than the heart [189].

Rong and Zhu suggested a common biological connection between the cardiac sympathetic nerve and the heart meridian (HM) [190]. Their findings provide a possible morphological and physiological explanation for the relation between referred cardiac pain and HM [190]. It is thus not surprising that traditionally, HM is being used to treat cardiac related illness, such as palpitation or cardiac arrhythmia, coronary heart disease, angina pectoris, myocardial infarction, ischaemic heart disease, cardiovascular disease and hypercholesterolemia [144,148,150,151,191,192,193,194].

## 7. Qi and Energy Fields

In the theory of TCM, Qi is thought to be a form of balancing energy that flows through the body. Qi keeps the body in harmony with the internal and the external environment that brings together the overall wellbeing of a person [126,129,142,143,144,150].

Chapter 18 of Huang Di Nei Jing Ling Shu [127], and Maciocia [192], give a detailed explanation about Qi formation. The production of Qi comes from the combination of food that we take and air that we breathe. Food is digested by the stomach and the nutrition is absorbed by the spleen and sent to the lung to form gathering Qi. The gathering Qi assists the lung in controlling Qi and breathing, and enhances the heart’s function of governing blood and blood vessels. It combines with original Qi that originates from the kidney and can be compared to the dynamic motive force that activates and moves the functional activity of all the organs, to form true Qi. True Qi is the final stage in the process of refinement and transformation of Qi. True Qi is further divided into nutritive Qi and defensive Qi. The main function of nutritive Qi is to nourish the internal organs. Nutritive Qi is also related to blood, and flows in the channels and blood vessels. Nutritive Qi is activated whenever a needle is inserted in an acupuncture point. As the name implies, defensive Qi means to defend or to protect the body. Unlike nutritive Qi, it flows outside of the channels [192].

### 7.1. How to Relate Qi to Molecular Knowledge of Modern Medicine?

In modern medicine, energy is the product of metabolic degradation of food and nutrients. The process of energy formation involves a complex series of various types of chemicals, molecules and cellular reactions. The process of metabolism is a collection of chemical catabolic reactions that break down the food we eat, from protein into amino acids, fats into fatty acids, and carbohydrates into glucose by the enzymes of the digestive system. In a nutshell, the nutrients from food are converted into adenosine triphosphate (ATP), which provides the basic unit of energy required for all functions in the body. A molecule of ATP consists of adenine, ribose, and three phosphate groups. Energy together with adenosine diphosphate (ADP) and a phosphate group (symbolized as Ⓟ) are released when the terminal phosphate group is split off ATP. About 40% of the energy released in metabolism is used for cellular functions. The rest is converted to heat, some of which helps with maintaining the body temperature [188]. ATP is also involved in the process of muscle contraction and relaxation [188], inflammation [102] and the analgesic effect of acupuncture [195,196,197,198,199,200,201], as explained below.

### 7.2. Energy and Information

Albert Szent-Györgyi, was a Hungarian biochemist who discovered vitamin C (deficiency of vitamin C from malnutrition is the main cause of scurvy [131]) and won the Nobel Prize in Physiology or Medicine in 1937. He quoted “In every culture and in every medical tradition before ours, healing was accomplished by moving energy” [202]. The word “energy”, for Szent-Györgyi, meant a cloud of electrons held together by nuclei [203]. He published a trilogy [202,204,205], based on his observation in the presence of energy in the living organisms during his study on cancer research. His study of energy in biology was linked to the role of purines and ATP as extracellular signaling molecules [206]. Later, this would have a great impact on the research of acupuncture. James Oschman, author of Energy Medicine, described the involvement of energy in the healing processes. The body is capable to take in, store, release, conduct, and utilise various kinds of energy and information [207].

Energy and information are also involved in the process of intercellular communication. This is done through action potential, an electrical signal conduction transporting information from one neuron to the next [102]. For example, action potential takes place by sending electrical impulses to the brain through afferent nerve fibres, such as in the event of pain or injury. To carry the information from one neuron to the next, neurotransmitters require energy in the form of ATP [208]. An action potential is also triggered by the stimulation of acupuncture needle insertion, which in turn affects the activities in the brain neuronal network [209].

Robert Becker, in his book, The Body Electric [210], hypothesizes that the acupuncture meridians are electrical conductors that send injury signals to the brain, which responds with the appropriate level of direct current to stimulate healing in the injured area. He also believed that the conductivity of the skin is much higher at acupuncture points. 

Björn Nordenström, a Swedish radiologist and surgeon, and the author of Biologically Closed Electric Circuits [211], believed that Qi is equivalent to or perhaps the same as the electromagnetic energy found in biologically closed electrical circuits (BCEC). Its Yin and Yang components may be compared to the positive and negative electrical charges of closed circuit ionic flow. If the acupuncture needle is inserted through the normal muscle into the injured muscle, it thus acts as a short circuit to enable the flow of ions. This facilitates healing of the injured tissue. Both Becker [210] and Nordenström [211] believed that the process of healing might work electrically.

In 1980, Cohen et al. measured the magnetic fields produced by the human body with the sensitive magnetic detector called the Superconducting Quantum Interference Device (SQUID) [212]. The body’s fluctuating magnetic fields, such as those from the heart are 100 times stronger than the field generated by the brain and can be detected up to a meter away from the body, in all directions, using SQUID-based magnetometers [213]. Both the magnetic fields produced by the heart and brain, can be measured via electrocardiogram (ECG) and electroencephalogram (EEG), respectively. Russek et al. proposed that the heart plays the major role in generating as well as integrating the flow of energy in the body [214].

Probably one of the best possible ways to explain the nature of Qi in a scientific manner is to relate it to the theory of extracellular and intracellular signaling. Qi can be considered to derive from signaling processes in the body, which include phosphorylation/dephosphorylation cascades, and guanine nucleotide-binding proteins (G protein) signaling involving cyclic adenosine monophosphate (cAMP) production and degradation, or calcium release and sequestration [215]. The balance between Yin and Yang that constitutes Qi could reflect cellular activities, which balance the actions of endogenous agonists and antagonists, and the sympathetic and parasympathetic nervous systems [216].

## 8. Is Acupuncture a Placebo Intervention?

Blinded controlled studies have addressed the following question. Do contextual effect and/or placebo acupuncture needle have the same therapeutic outcome as real acupuncture? The results from many randomized controlled trials (RCTs) have both proved and disproved the benefit of acupuncture. As compared to other types of pharmacological RCTs, the study of acupuncture is very complex due to the involvement of many issues, such as inadequate sample size, randomization, sham interventions, and blinding. The tendency that potential bias might arise from both the sham interventions and blinding procedures is another great challenge in such studies. Patients’ expectation may also influence the final outcomes of the trials [217]. Table 1 summarized some of the RCTs of LBP treated with acupuncture. There was no special search strategy used to select the publications for Table 1 and Table 2 and therefore it is rather an overview than evidence based on a systematic search method. Most articles can be found in the database Pubmed.

### 8.1. In the Wake of Double-Blind Randomised Control Trials (RCTs)

The double-blind placebo-controlled clinical trial is considered as the gold standard for testing or comparing the efficacy of either new or existing treatments/drugs [233]. The idea was put forward by Austin Bradford Hill back in 1937 [234]. Before the mid-20th century, the practice of informed consent was not mandatory. Patients were often not informed of their involvement in a clinical study and did not realize that they might receive a placebo instead of a real treatment [235]. 

The main purpose to conduct a clinical RCT is to prevent bias or manipulation of results in medical studies [217,234,235,236]. Such biases originate from several sources. Patients’ awareness of trials might lead to exaggerated expectations and responses [217,235,237,238,239,240,241,242,243,244,245,246,247]. Some clinical researchers might depend heavily on the pharmaceutical industry funding and may not be effectively protected from transmitting their sponsor’s bias in favour of a new drug [247,248]. Surveys showed that double-blind methodologies are still not always properly applied to clinical tests [236,249,250,251].

### 8.2. The Placebo Effect

The question of whether acupuncture merely produces a placebo effect has to be considered in light of numerous studies done in this field. The word “placebo”, is a Latin word, usually translated as “I shall please” [237]. According to Shapiro, a placebo is defined as “any therapeutic procedure (or a component of any therapeutic procedure) which is given (1) deliberately to have an effect, or (2) unknowingly and has an effect on a symptom, syndrome, disease, or patient but which is objectively without specific activity for the condition being treated. The placebo is also used as an adequate control in research. The placebo effect is defined as the changes produced by placebos” [252].

As stated by Walter Brown, author of The Placebo Effect in Clinical Practice, the act of seeking and receiving medical care itself is a placebo effect. Some conditions are highly placebo-responsive, which could bring up to as much as 50% relief. The placebo treatment can be used to enhance the benefits of standard treatments. The healing environment, which involves the personal attention and the two-way communication between a patient and a care provider, is a powerful antidote for illness [235]. Miller and Kaptchuk proposed this kind of placebo effect as “contextual healing”, in which a specific clinical encounter contributes to therapeutic outcomes [253]. Jay Katz, author of The Silent World of Doctor and Patient, noted that “Physicians and patients may gradually learn that the placebo effect is an integral and inevitable component of the practice of medicine, that it constitutes its art and augments its science” [254]. Kerr et al. expressed “neurophenomenological” as the reason that caused the placebo effect from sham acupuncture due to the palpation and touch by the practitioner and tactile stimulation by sham acupuncture needles [255].

The mechanisms of the placebo analgesic effects include activation of endogenous opioids and dopamine release, and alteration of central processing of pain [238,239,240]. Expectation of benefit also plays an important role in the efficacy of placebo interventions [238,239,240,241,242,243,244,245]. Henry Beecher in his article “The Powerful Placebo”, suggests that placebos can relieve pain arising from physiological causes and have an average significant effectiveness of 35.2 ± 2.2% [256].

### 8.3. Clinical Studies

The placebo effect produced by sham acupuncture, derives from patients’ expectation and bias. Yet, this does not mean that the therapeutic effect of acupuncture is only explained as a placebo intervention. In comparing placebo to no treatment, such as those shown in Table 1, placebo was significantly more effective than no-treatment groups or even standard medical care. This was demonstrated by an impressive series of three-arm studies in German Acupuncture Trials (GERAD) for relief of LBP [223]. Subsequently, the outcome of treatment of chronic LBP by acupuncture was so positive that German insurance companies agreed to cover the cost of such treatments [223,257].

Therefore, it is safe to say that a placebo intervention that produces a real effect is not supposed to be called a “placebo”. There must be some physiological or psychological mechanism that explains such effects. Besides tactile stimulation [255], several studies proposed that afferent nerve fibres are involved in response to acupuncture treatment [110,112]. 

### 8.4. Acupuncture Points for Lower Back Pain

In Table 2, only six out of 15 RCTs stated precisely which acupuncture points were used for the treatment of LBP. Of these, the most commonly used acupuncture points are GV 3, BL 23, BL 25 and BL 40. GV 3, BL 23 and BL 25 are all local points, while BL 40, located in the popliteal fossa, is a distal point for treating LBP. Other distal points are BL 60 and K 3, they are situated behind the lateral and medial malleolus.

The most commonly used acupuncture points in the treatment of LBP are listed in the literature [232,258]. Lee et al. did an investigation based on 53 clinical studies and came out with a summary of the most frequently used acupuncture points for the treatment of LBP [258]. It clearly shows that both the bladder and gallbladder meridians are the chosen choice for treating LBP with acupuncture, especially BL 23 (51%), follow by BL 25 (43%), BL 24, BL 40, BL 60 and GB 30 (32% each), BL 26 and BL 32 (28% each), and GB 34 (21%). In the opinion of Maciocia, the proper selection of local and distal points is based on the location and the nature of pain, and depends on the condition, if it is either acute or chronic [144].

### 8.5. Clinical Relevance

According to Robinson [176], acupuncture points BL 23, BL 24 and BL 26 improve circulation to the local tissues and resolve myofascial dysfunction and promote tissue recovery. BL 25 is stimulated to relax the myofascia and free the nerves which are entrapped and irritated. BL 40 may improve circulation and nerve health by reducing myofascial tension caused by neurovascular compression. BL 60 and K 3 are located on each side of the ankle, and represent the distant points for LBP. They contain an extensive sympathetic nerve supply that provides homeostatic effects. GB 30 sits on top of the sciatic nerve and is a good choice to treat sciatica caused by piriformis entrapment syndromes. GB 34, located on the neck of fibular, is a choice point to treat common peroneal (fibular) nerve syndrome. GV 3, which is supplied by the spinal segmental nerve and the somatosomatic as well as somatovisceral reflex connections, has neuromodulatory properties for treating LBP, paraparesis, sciatic pain, genitourinary conditions, and lower gastrointestinal disorders.

## 9. Mechanism of Acupuncture

What is actually happening the moment an acupuncture needle penetrates the skin [259]? According to Robinson, this interesting phenomenon is due to a higher density of sensory and sympathetic nerve fibres attached to blood vessels in distal than in proximal segments. Such an anatomical arrangement accounts for the relatively stronger autonomous responses associated with distal acupuncture points, especially those at the ends of the channels [176]. Dellon et al. came to the same conclusion concerning the higher concentration of sympathetic innervation in the foot [260].

Manipulation of acupuncture needle results in deformation of connective tissues and thus alters the structure of fibroblasts [197,261,262,263,264]. Such micro-injury caused by the puncturing of the acupuncture needle in the skin results in release of ATP [195,196,197,198,199,200,201,265,266]. ATP is then further broken down into adenosine and other purines [195,196,197,198,199,200,201,265,266]. Both ATP and adenosine act as anti-nociception agents that block pain through purinergic receptors [195,196,197,198,199,265,266,267,268,269]. 

### 9.1. Deqi

The pricking sensation caused by stimulation of acupuncture needle is termed Deqi. This refers to the excitation of Qi or vital energy inside meridians. Often patients might experience a combination of various sensations, including numbness, soreness, distention, heaviness, dull pain, or sharp pain during acupuncture needle insertion [270]. Deqi, which was mentioned in the chapter 1 of Huang Di Nei Jing Ling Shu [127], literally means “the arrival of vital energy”, which is widely viewed as an important role in the process of achieving therapeutic effectiveness of acupuncture treatment [127,165,271,272,273,274]. A functional magnetic resonance imaging (fMRI) study conducted by Napadow et al. shows an increase of blood flow to the penetrated sites that are linked to the sensation of Deqi [275]. Kong et al. found that the different intensity of acupuncture sensations is associated with the effect of analgesia [276]. Spaeth et al. concluded that real acupuncture tends to produce a stronger Deqi sensation and better clinical outcomes as compared to sham acupuncture [277]. However, White et al. indicated that the sensation of Deqi does not impact the efficacy of acupuncture treatment [278].

### 9.2. Needle Grasp

The phenomenon of needle grasp is described as a tug on the needle, like a fish biting on a fishing line, creating a tight mechanical coupling between needle and tissue. Langevin et al. proposed that needle grasp perceived by acupuncturists corresponds to the sensation of Deqi, which is felt by patients. This may play an active role in the therapeutic mechanism of acupuncture [159,279,280]. These authors hypothesize that the manoeuvre of the needle (by rotation or piston movement) causes needle grasp, and hence, Deqi is a result of collagen and elastic fibres winding and tightening around the needle. Such a mechanism is responsible for the increase in pull-out force induced by needle rotation [159,279,280,281]. The higher density of connective tissues at acupuncture points might explain the occurrence of needle grasp [159]. 

Needle grasp allows further movements of the needle to pull and deform the connective tissue surrounding the needle, delivering some of its effects through mechanical influence on the connective tissue matrix [159,279,280,281]. In addition, the manipulation of the acupuncture needle causes remodeling of fibroblasts in the connective tissue [261,262,263,264].

### 9.3. Afferent Nerve Fibres

Many RCT studies claimed the lack of significant difference between real and sham acupuncture (Table 1) [224,227,231,232]. This conclusion arose from the fact that both real and sham acupuncture in these RCTs are involved in tactile stimulation [255]. The reason behind such phenomenon is due to the stimulation of the afferent nerve fibres [110,112,282]. Such superficial pressure via sham needling intervention can have significant effects on the brain’s limbic system [245,283]. Acupuncture fMRI studies have shown that both tactile stimulation and acupuncture manipulation activate neural activities and thus might have a potential effect on pain modulation [283,284,285,286].

The human tactile sensation is thought to be moderated primarily by large myelinated afferents (Aα and Aβ), while pain and temperature sensations are brought about by small myelinated (Aδ fibre) and unmyelinated (C fibre) afferents [287]. It is therefore plausible that many sham acupunctures, used as control procedures in acupuncture studies, activated this group of afferents and hence produced analgesic effects [110,112]. Zhou et al. concluded that afferent fibres of type II (Aβ) and III (Aδ) are responsible for transmitting the acupuncture signal, which are important for acupuncture analgesia [168].

### 9.4. Acupuncture Analgesic

The study of acupuncture in pain management and its analgesic effects have been highlighted in the literature [110,112,119,134,136,137,138,139,140,142,143,144,146,147,151,163,170,176,192,195,196,197,198,199,200,201,235,241,288,289,290]. The gate control theory of pain, which was proposed by Melzack and Wall back in 1965 [93], and later by Melzack, suggested that the stimulation by the acupuncture needle activates the inhibitory brainstem system and therefore blocks pain signals [291]. The release of endogenous opioids [112,119,163,292] and ATP [195,196,197,198,199,200,201,265,266] triggered by an acupuncture needle have been well documented by many studies. This suggests a link between acupuncture and signaling mediated by neurotransmitters.

Endorphins are amongst the most studied neurotransmitters in acupuncture research. They are more powerful in pain relief than exogenous morphine [293]. A recent study conducted by Grissa et al. compared the effectiveness of acupuncture versus morphine in the management of acute pain in the emergency department. A reduction of pain by 92% was observed in patients treated with acupuncture, while morphine-treated patients experienced only 78% reduction. In addition, acupuncture-treated patients had a much faster pain recovery time (an average of 16 min compared to 28 min in the morphine control group) [294].

### 9.5. ATP as Neurotransmitter

Adenosine triphosphate (ATP) was discovered by Karl Lohman in 1929 [295]. In that same year, Drury and Szent-Györgyi described the effects of ATP on heart excitability, lowering of blood pressure and coronary vasodilation [206]. Research by Holton and Holton in 1954 identified ATP as a neurotransmitter in the somatosensory system. They demonstrated that ATP is released from peripheral endings of primary sensory neurons [296]. The signaling function of ATP in peripheral tissues was confirmed by numerous investigations done by Geoffrey Burnstock. This led to the discovery of Purinergic Signaling that consists of neurotransmission by purinergic receptors [297].

In 2009, Burnstock proposed that stimulation of the tissue with acupuncture needle, heat or electrical current triggers the release of a large quantity of ATP from keratinocytes, fibroblasts and other cells in the skin [298]. ATP activates P2X ligand-gated ion channel 3 receptors (P2X3), which are located on sensory nerves. Resulting signals modulate the pathways that lead to the CNS responsible for conscious awareness of pain [265,266,267,268,299,300,301,302]. 

### 9.6. Adenosine-Induced Anti-Nociception

As mentioned before, energy in the form of ATP is one of the essential requirements for our body’s function [188]. Adenosine, which is the core molecule of ATP and of nucleic acids, forms a unique link among cell energy, gene regulation, and neuronal excitability [302]. Adenosine is recognised by specific receptors, which regulate neuronal and non-neuronal cellular functions. As a neurotransmitter, adenosine regulates pain transmission in both the spinal cord and in the periphery [303]. Adenosine acts as an endogenous anti-inflammatory agent and as such plays an important role as a signaling molecule in immunity and inflammation [304]. Hence, adenosine is involved in nearly every aspect of cell function.

Recently, in a series of experiments by Goldman et al., adenosine was identified as a mediator of anti-nociceptive properties in acupuncture experiments in mice [195]. In another study conducted by the same group in human subjects, they used tissue microdialysis to demonstrate the increased release of adenosine, adenosine monophosphate (AMP), adenosine diphosphate (ADP), and ATP at stimulated acupuncture points [196]. Both studies show that the concentration of interstitial purines were increased by mechanical stimulation from the acupuncture needle. In contrast, transgenic mice lacking adenosine A1 receptors do not show any sign of pain reduction [195].

Pharmacologically, the anti-nociceptive effect of acupuncture is prolonged by 2 to 2.5 h when the combination of acupuncture and injection of deoxycoformycin (dCF) were introduced [195,199]. Hurt and Zylka suggested that a longer period of pain relief might be achieved by injecting prostatic acid phosphatase (PAP) into the acupuncture points [305]. Both dCF and PAP are agonists of ATP production in response to acupuncture treatment [195,305]. But the opposite is observed when an adenosine receptor antagonist, such as caffeine, is either taken orally or injected at the acupuncture points, which then interfere with the analgesic effect of acupuncture [199,306]. Interestingly, from their observation, Tang et al. concluded that the higher efficacy of acupuncture in China than in western countries could be due to the lower consumption of coffee in China than in Europe and America [268]. 

## 10. Discussion

Low back pain is one of the most common musculoskeletal disorders in modern society [1,2,3,4]. There are increasing numbers of patients seeking complementary therapies, such as acupuncture, as a mean to supplement the conventional treatments. Many studies have produced conflicting results relating to the efficacy of acupuncture as a method to treat LBP. This is probably due to a small sample sizes, a lack of blinding procedures and improper methodological assessment tools [5,61,65,113,115].

In most RCTs, sham acupuncture was used as a control, either using sham acupuncture needles which do not penetrate the skin, or selecting sham acupuncture points (Table 1). It was interesting to observe the outcomes from the verum and sham acupuncture groups (Table 1). Both groups produced better results than conventional therapies or non-treatment groups and it was obvious that verum acupuncture produced slightly better results than sham acupuncture. The reason behind such a phenomenon could be attributed to tactile stimulation. Both puncturing the skin by acupuncture needle, and stimulation by sham needle on the skin surface, activate the afferent sensory receptors, leading to therapeutic effects.

The high density of nerve endings under acupuncture points could be another reason that contributed to the acupuncture-induced analgesic effects, which yields better results in verum than in the sham acupuncture group. The release of purines, such as ATP and adenosine, which bind to the purinergic receptors, provide a hypothesis to explain the efficacy in acupuncture against pain [110,112,119,134,136,137,138,139,140,142,143,144,146,147,151,163,170,176,192,195,196,197,198,199,200,201,235,241,288,289,290].

Why is acupuncture effective on some patients, but not on others? Many factors could give rise to such uneven responses. Patients’ expectations might play a crucial role in the determination of the outcome of an acupuncture trial. So does the tendency of researchers’ strong bias in favour of or against the efficacy of acupuncture, which might affect the overall outcome of a trial. 

Combination of acupuncture with other regular therapies produces better results than conventional therapies alone. Therefore, a holistic approach to integrate acupuncture with other types of conventional treatments should be carried out for the benefit of the patients.

Of course our article also has potential limitations. In addition to the mentioned literature, many other high-quality RCTs on acupuncture and LBP have been conducted recently [307,308,309]. Although, the meta-analysis results of some of the systematic reviews indicated that acupuncture might be an effective treatment for chronic LBP in the short-term, conclusions among the reviews are inconsistent overall.

## 11. Conclusions

The WHO confirmed in 2002, the effectiveness of acupuncture treatment from controlled clinical trials for 28 diseases, symptoms, and conditions. LBP was one of the conditions that was mentioned in this report [310]. A meta-analysis of 33 RCTs of acupuncture for LBP showed better results than sham acupuncture and no treatment [311]. Meanwhile, the evidence for efficacy of the German Acupuncture Trials (GERAD) of 1162 patients with chronic low back pain compared verum acupuncture, sham acupuncture and a conventional therapy group as the control. The effectiveness of acupuncture, both verum and sham, was almost twice that of conventional therapy 6 months after the trial [223]. Acupuncture is recommended for patients with chronic LBP due to its cost-effectiveness [312] and low adverse effects [313].

The discovery of purines-mediated (both ATP and adenosine) anti-nociceptive effects of acupuncture, has led to a better understanding of molecular events underlying the mechanism of acupuncture in the peripheral nervous system. However, our knowledge of mechanisms associated with acupuncture’s analgesic properties remains limited. Integration of acupuncture treatments with conventional therapies, and pharmacological agents should lead to medical strategies without addictive side effects. 

Despite shown effectiveness by RCTs and the discovery of potential molecular mechanisms, acupuncture is still not well accepted by the social security services in Austria and many European countries. Such a situation results from health policy and the regulation of the healthcare bodies in different European Union (EU) members. Yet, any treatment producing better outcomes at a lower cost is a desirable step towards more sustainable health systems.

## Figures and Tables

**Table 1:** Results of randomized control trials for acupuncture treatment of lower back pain.

Authors	Diagnosis	Intervention Group	Control Group	Outcome Measure	Result
Pach et al. (2013) [218]	CLBP	*n* = 73, standardized manual acupuncture; *n* = 66, individualized manual acupuncture	NA	VAS	Both intervention groups showed improvement in pain scale but there were no relevant difference between them
Molsberger et al. (2002) [219]	LBP	*n* = 65, manual acupuncture + conventional orthopaedic therapy	*n* = 61, sham acupuncture + conventional orthopaedic therapy; *n* = 60, conventional orthopaedic therapy	VAS	Acupuncture + conventional orthopaedic therapy were better than sham and conventional orthopaedic therapy alone
Weiß et al. (2013) [220]	CLBP	*n* = 74, manual acupuncture + inpatient rehabilitation program	*n* = 69, inpatient rehabilitation program	SF-36	Intervention group showed better results judging from SF-36 questionnaires
Inoue et al. (2006) [221]	LBP	*n* = 15, manual acupuncture	*n* = 16, sham acupuncture	VAS, Schober test	Both groups showed reduction in pain but intervention group showed better result than control group
Giles et al. (2003) [222]	CSP	*n* = 36, manual acupuncture; *n* = 36, spinal manipulation	*n* = 40, medication	ODI, NDI, SF-36, VAS	Manipulation achieved the best overall results, however, on the VAS for neck pain, acupuncture showed a better result than manipulation (50% vs. 42%)
Haake et al. (2007) [223]	CLBP	*n* = 387, manual acupuncture	*n* = 387, sham acupuncture; *n* = 388, conventional therapy (physiotherapy, exercise)	CPGS, HFAQ	Effectiveness of acupuncture, both verum and sham, was almost twice that of conventional therapy
Brinkhaus et a (2006) [224]	CLBP	*n* = 146, manual acupuncture; *n* = 73, minimal manual acupuncture	*n* = 79, waiting list	SF-36, VAS	Acupuncture was better than no acupuncture, but no significant differences between acupuncture and minimal acupuncture
Cho et al. (2013) [225]	CLBP	*n* = 57, manual acupuncture	*n* = 59, sham acupuncture	VAS	Acupuncture was better than sham acupuncture
Cherkin et al. (2001) [226]	CLBP	*n* = 94, manual acupuncture	*n* = 78, massage; *n* = 90, self-care	SBS, RDS	Massage was better than acupuncture and self-care
Cherkin et al. (2009) [227]	CLBP	*n* = 158, standardized manual acupuncture; *n* = 157, individualized manual acupuncture; *n* = 162, simulated acupuncture (using toothpick)	*n* = 161, usual care (medications, physiotherap)	RMDQ	All intervention groups showed better outcome than usual care, but no significant differences among the acupuncture groups
Yun et al. (2012) [228]	CLBP	*n* = 82, standardized manual acupuncture; *n* = 80, individualized manual acupuncture	*n* = 74, usual care (massage, physiotherapy, medications)	RMDQ, VAS	Intervention groups showed better results than control; but individualized acupuncture is more effective than standardized acupuncture
Zhang et al. (2017) [229]	DiscogenicSciatica	*n* = 50, 50 Hz electroacupuncture	*n* = 50, MFE	NRS, ODI, PGI	The effect of electroacupuncture was superior to that of MFE
Thomas et al. (1994) [230]	CNLBP	*n* = 7, manual acupuncture; *n* = 9, 2 Hz low frequency electroacupuncture; *n* = 11, 80 Hz high frequency electroacupuncture	*n* = 10, waiting list	ADL related to pain, ROM	All intervention groups showed reduction of pain, more so in low frequency electroacupuncture group in long term
Glazov et al. (2014) [231]	NSCLBP	840 nm laser acupuncture: *n* = 48, 0.8 Joules high dose; *n* = 48, 0.2 Joules low dose	*n* = 48, 0 Joules sham laser acupuncture (without switching on the laser)	NPRS, ODI	Treatment groups showed better result but no difference between sham and laser groups
Shin et al. (2015) [232]	LBP	660 nm laser acupuncture: *n* = 28	*n* = 27, sham laser acupuncture (without switching on the laser)	VAS, PPT	Both groups showed improvement in pain but no significant difference outcomes between the two groups

NA = Not Available; ADL = Activities of Daily Life; CPGS = Chronic Pain Grade Scale; HFAQ = Hanover Functional Ability Questionnaire; MFE = Medium-Frequency Electrotherapy; NDI = Neck Disability Index; NRS = Numerical Rating Scale; ODI = Oswestry Disability Index; NPRS = Numerical Pain Rating Scale; PGI = patient global impression; PPT = Pressure Pain Threshold; RDS = Roland Disability Scale; RMDQ = Roland-Morris Disability Questionnaire; ROM = Range of Motion; SBS = Symptom Bothersomeness Scale; SF-36 = Short-Form 36 Health Survey; VAS = Visual Analog Scale; CLBP = chronic low back pain; CNLBP = chronic nociceptive low back pain; CSP = chronic spinal pain; LBP = low back pain; NSCLBP = non-specific chronic low back pain; nm = Nanometer.

**Table 2:** Selection of acupuncture points of randomized control trials for treatment of lower back pain.

Authors	Local Points	Distant Points	Other Points
Pach et al. (2013) [218]	BL 23, 24, 25	BL 40, 60; GB 34; K 3	-
Molsberger et al. (2002) [219]	BL 23, 25; GB 30	BL 40, 60; GB 34	4 Ashi Points of maximum pain
Weiß et al. (2013) [220]	NA	NA	-
Inoue et al. (2006) [221]	-	-	Single Ashi Point at the most painful point
Giles et al. (2003) [222]	8 to 10 needles were placed in local paraspinal intramuscular maximum pain areas	Approximately 5 needles were placed in distal acupuncture point	-
Haake et al. (2007) [223]	14 to 20 needles were inserted but exact locations were not mentioned		
Brinkhaus et al. (2006) [224]	At least 4 local points: BL 20 to 34; BL 50 to 54; GB 30; GV 3, 4, 5, 6	At least 2 distant points: SI 3; BL 40, 60, 62; K 3, 7; GB 31, 34, 41; LR 3; GV 14, 20	Extraordinary Points: Huatojiaji & Shiqizhuixia
Cho et al. (2013) [225]	Points were chosen according to 3 types of meridian patterns: 1. Gallbladder Meridian: 12, 26, 30, 34, 41 2. Bladder Meridian: 23, 24, 25, 37, 40 3. Mixed Meridian: ST 4, 36; SP 13, 14; GV 3, 4, 5, 24, 26		
Cherkin et al. (2001) [226]	NA	NA	-
Cherkin et al. (2009) [227]	1. Individualized Acupuncture: Averaged of 10.8 needles, chosen from 74 points, half from Bladder meridian 2. Standardized Acupuncture: 8 acupuncture points commonly used for chronic low back pain (GV 3, BL 23 *, BL 40 *, K 3 *, Low Back Ashi Point) 3. Simulated Acupuncture: Using toothpick on acupuncture points		
Yun et al. (2012) [228]	GV 3; BL 23	BL 40; K 3	Low Back Ashi Points, Back-Pain Points ^
Zhang et al. (2017) [229]	BL 25	-	Extraordinary Points: JiaJi * (Ex-B2)
Thomas et al. (1994) [230]	BL 23, 25, 26, 32; GB 30, 34	BL 40, 60; SI 6; ST 36	-
Glazov et al. (2014) [231]	An average of 9 points were used: GV 13%, BL 37%, GB 13%, other meridians 16%, Ashi Points 14%, Extraordinary Points 7%		
Shin et al. (2015) [232]	GV 3, 4, 5; BL 23 *, 24 *, 25 *; GB 30 *	BL 40 *	-

BL = Bladder; GB = Gallbladder; GV = Governor Vessel; K = Kidney; LR = Liver; SP = Spleen; ST = Stomach; NA = Not Available; * = Bilaterally; ^ = Extra Meridian points on the back of hand.

## Abbreviations

5-HT	5-hydroxytryptamine
A1R	Adenosine A1 receptor
ADL	activities of daily life
ADP	adenosine diphosphate
AIDS	acquired immune deficiency syndrome
AMP	adenosine monophosphate
ATP	adenosine triphosphate
BCEC	Biologically Closed Electric Circuits
BMI	body mass index
cAMP	cyclic adenosine monophosphate
CGRP	calcitonin gene-related peptide
CLBP	chronic low back pain
CNLBP	chronic nociceptive low back pain
CNS	central nervous system
CO_2_	carbon dioxide
COPD	chronic obstructive pulmonary disease
CSP	chronic spinal pain
CVDs	cardiovascular diseases
DALYs	disability-adjusted life years
dCF	deoxycoformycin
DRG	dorsal root ganglia
EA	electroacupuncture
ECG	electrocardiogram
EEG	electroencephalogram
EU	European Union
fMRI	functional magnetic resonance imaging
GBD	The Global Burden of Disease Study
GERAD	German Acupuncture Trials
GIDs	gastrointestinal disorders
G proteins	guanine nucleotide-binding proteins
GV	Governing Vessel
H^+^	protons
H_2_O	water
HIV	human immunodeficiency virus
HM	Heart meridian
LBP	lower back pain
MA	manual acupuncture
MFE	Medium-Frequency Electrotherapy
mTrPs	Myofascial Trigger Points
MUS	medically unexplained symptoms
NGF	nerve growth factor
NRS	numerical rating scale
NSAIDs	non-steroidal anti-inflammatory drugs
NSCLBP	non-specific chronic low back pain
P2X3	P2X ligand-gated ion channel 3 receptor
PAP	prostatic acid phosphatase
PGI	patient global impression
PN	primo node
PPT	pressure pain threshold
PV	primo vessel
PVS	primo vascular system
RCTs	randomised control trials
ROM	range of motion
SQID	Superconducting Quantum Interference Device
TCM	Traditional Chinese Medicine
TENS	transcutaneous electric nerve stimulation
TRP	transient receptor potential
TRPA1	transient receptor potential ankyrin 1
TRPV1	transient receptor potential vanilloid 1
VAS	visual analogue scale
WHO	World Health Organisation
YLDs	years lived with disability
